# Mental health help-seeking in New York City: How intersecting identities shape informal and formal support use

**DOI:** 10.1371/journal.pone.0356292

**Published:** 2026-08-14

**Authors:** Thinh Toan Vu, Jo-Anne Caton, Christina Norman

**Affiliations:** Bureau of Mental Health, New York City Department of Health and Mental Hygiene, New York, United States of America; Universidad Técnica de Manabí: Universidad Tecnica de Manabi, ECUADOR

## Abstract

**Introduction:**

This study investigated how intersecting identities, including race and ethnicity, gender, socioeconomic status, and immigration-related characteristics, impact help-seeking for emotions, nerves, or mental health among New York City (NYC) adults. It also identified primary sources of informal and formal support.

**Methods:**

Using a citywide representative sample of 43,606 non-institutionalized adults, data were collected between May and September 2023. Log-linear regression models assessed main effects and conditional effects (i.e., interactions) of sociodemographic and immigration-related characteristics on mental health help-seeking in the past 12 months. Predicted probabilities from models with significant interaction terms illustrated intersectional impacts. Sensitivity analyses among participants with past 30-day moderate-to-high psychological distress tested robustness of these findings.

**Results:**

Approximately 46.6% of adults sought any mental health support, with 36.2% seeking informal support and 24.2% seeking formal support. Family/friend (33.1%) and therapist/counselor (16.8%) were the most common informal and formal sources, respectively. Significant interactions for any help-seeking emerged between race and ethnicity with gender (p = 0.003), employment status (p < 0.001), place of birth (p < 0.001), and English proficiency (p < 0.001). These interactions held for both formal and informal support, except there was no significant interaction with gender for informal support nor with English proficiency for formal support. Sensitivity analysis among those with past 30-day moderate-to-high psychological distress produced similar findings compared to all adults.

**Conclusions:**

Mental health help-seeking in NYC varies significantly across intersecting social identities, highlighting disparities linked to race and ethnicity, gender, immigration status, and language proficiency. Addressing these disparities requires improving accessibility and cultural relevance of formal mental health services while recognizing and strengthening trusted informal support systems.

## Introduction

Mental health continues to be a pressing public health concern in the U.S., including in New York City (NYC), where 22.9% of adults reported experiencing any mental illness in the past year. Despite this high burden, more than half of adults with any mental illness did not receive treatment, and over a fifth perceived an unmet need for mental health treatment [1]. Left unaddressed, such unmet treatment needs are projected to contribute to an estimated US$14 trillion in excess costs nationally by 2040 [2]. Although experiences with mental health challenges vary, the process of seeking help, whether formal (from health professionals) or informal (from family, friends, or community networks), is a beneficial coping strategy. Help-seeking is not limited to those with a clinical diagnosis; it also encompasses the everyday reasons people seek support, advice, or relief in response to emotional distress, life stressors, or uncertainty (e.g., job changes, relationship strain, or unwanted habits) [3]. In this broader context, mental health help-seeking serves as an adaptive coping mechanism [4] that can alleviate illness burden, lower treatment costs, and enhance overall functioning [5].

Mental health help-seeking is not solely an individual decision [6], it is deeply shaped by social and structural conditions. Specifically, help-seeking is significantly influenced by social determinants of mental health [7], the conditions into which people are born and in which they grow, live, work, and age. Adults from minoritized racial and ethnic groups may be less likely to seek help for mental health concerns in the first place, due to stigma, lack of culturally competent care, and mistrust of medical institutions [8]. Among those who do seek help, these groups remain consistently less likely than white counterparts to receive mental health services [9–11]. In NYC, non-Hispanic white adults with serious psychological distress (58%) are more likely to have received past-year mental health treatment compared to non-Hispanic Black (48%), and non-Hispanic Asian (39%) adults [12], which may highlight racial and ethnic disparities in mental health help-seeking. Gender disparities also persist, with men less likely than women to seek mental health support [10,13–15]. Among NYC adults with any mental illness, approximately 47% of males and 53% of females received past-year mental health treatment [12], a pattern often attributed to traditional gender norms that discourage emotional expression and promote self-reliance, which may contribute to underutilization of services among men [16].

Socioeconomic status represents another important social determinant of mental health help-seeking [17,18]. Individuals experiencing poverty or financial hardship often face significant barriers to seeking professional mental health services. During the COVID-19 pandemic, NYC adults living in low-income housing encountered twice the obstacles in seeking mental health services (e.g., obtaining health insurance, stigma, or low trust in providers) compared to their counterparts residing in market-rate housing [19]. Immigration-related factors add another layer of complexity. Immigrants often encounter unique barriers to care, including language difficulties, unfamiliarity with the healthcare system, and fears related to immigration status, which could discourage or delay help-seeking [20]. Place of birth, English proficiency, and residence duration in the U.S. have all been shown to influence mental health outcomes and help-seeking patterns. Particularly, individuals who are foreign-born [20] or have limited English proficiency [15] are less likely to seek professional mental health services.

Intersectionality, a framework introduced over three decades ago [21] that takes into account intersecting identities such as one’s race and gender, offers a powerful lens for addressing these disparities [7]. It captures the complex, overlapping influences of social categories on individual and population health, rather than treating these factors as separate and additive [7,22]. While extensively applied in various fields, its integration into public health research is relatively recent [22]. In the U.S., health research has traditionally focused on documenting disparities in outcomes between racial and ethnic groups, often without examining how intersecting social identities shape these disparities [23]. Although the application of intersectionality in public health is increasing, studies often limit their analyses to a narrow range of identity combinations [22]. For instance, one study found that younger Black women were more likely than older Black women to use mobile technology to manage anxiety [24], but the focus on a single racial and gender group limited the broader applicability of the findings. While such studies yield important within-group insights [22], they may offer limited perspective on how multiple social identities interact across diverse populations. Without broader intersectional comparisons, it remains challenging to understand whether and why mental health help-seeking behaviors differ between groups, such as between Black women and women of other racial or ethnic backgrounds.

NYC offers a unique and timely context in which to explore these questions. As one of the most diverse urban cities in the world, NYC is home to a population that is 36.1% foreign-born, 50% racial or ethnic minority, and 17.9% living below the poverty line [25]. This exceptional demographic diversity presents a distinct opportunity to examine how intersecting social identities influence mental health help-seeking in a densely populated, highly heterogeneous, urban environment. To address the limitations of prior research, which has largely focused on formal mental health help-seeking and examined the individual effects of sociodemographic factors [20], this study had two aims. First, it investigated how the intersections of race and ethnicity with gender, socioeconomic status (education, employment, and poverty), and immigration-related characteristics (place of birth, English proficiency, and residence duration in the U.S.) influence both formal and informal help-seeking for issues related to emotions, nerves, or mental health among NYC adults. Second, the study identified the primary sources of mental health support, offering a comprehensive overview of health-seeking patterns.

## Materials and methods

### Study design and sample size

This population-based cross-sectional study utilized data from the 2023 Neighborhood Wellness Survey, NYC’s largest mental health survey to date. Full details on survey design, sampling procedures, and instrument development were described previously [26,27]. Briefly, adults aged ≥18 from all five boroughs were recruited through address-based sampling across all NYC ZIP codes to ensure a representative citywide sample. Surveys were mailed to 210,000 households between May and September 2023 and were available in five languages (English, Spanish, Haitian Creole, Russian, and Chinese), with a target sample size of 50,000 participants. A total of 52,802 surveys were returned. After excluding duplicates, respondents no longer living in NYC, questionnaires with <80% completion, and participants <18 years old, the final analytic sample included 43,606 participants.

### Measurements

**Mental health help-seeking:** was assessed as a dichotomous response (yes vs. no) to the question: “*In the past 12 months, did you seek support for your emotions, nerves, or mental health?*” Participants were also asked about the sources of mental health support they sought, categorized as either informal (i.e., a close family member/friend, spiritual or religious advisor, or someone else) or formal sources (i.e., family doctor or general practitioner; social worker, therapist, counselor, or psychologist; psychiatrist or other prescriber) [12]. These categories are not mutually exclusive, as some individuals may have sought both informal and formal support.

**Moderate-to-high psychological distress:** was defined as total score of eight or higher on the six-item Kessler psychological distress scale (range: 0–24), which measures symptoms like sadness, nervousness, and hopeless in the past 30 days [28].

**Lifetime mental and behavioral health diagnoses:** were categorized based on participants’ self-reported history across eight conditions: major depression or severe depression; post-traumatic stress disorder; anxiety or generalized anxiety disorder; schizophrenia, schizoaffective disorder, psychosis; bipolar disorder, mania, manic depression; intellectual or developmental disabilities (including Attention-Deficit/Hyperactivity Disorders [ADHD] and autism); drug use disorders (including cocaine, heroin, etc.); and alcohol use disorders.

**Sociodemographic characteristics:** included age groups (18–24, 25–44, 45–64, ≥ 65 years), gender (cisgender man, cisgender women, other gender identity), sexual orientation (straight/heterosexual, gay/lesbian/bisexual/some other orientations [LGB+], not sure), race and ethnicity (non-Hispanic white, non-Hispanic Black, non-Hispanic Asian, Hispanic, non-Hispanic, and non-Hispanic other including American Indian/Alaska Native, multiple races, and other; hereafter referred to as white, Black, Asian, Hispanic, and Other, respectively), marital status (single/never married, married, living with a partner, widowed, divorced/separated), educational level (high school or less, some college, bachelor or higher), health insurance (yes, no), employment status (employed, unemployed, not in labor force), and household poverty levels (<200% FPL, 200–399% FPL, ≥ 400% FPL).

**Immigration-related characteristics:** comprised place of birth (U.S.-born, foreign-born), English proficiency (not at all well/not well, well/very well), and residence duration in the U.S. (<5 years, 5–10 years, >10 years).

### Statistical analysis

Sociodemographic profiles were weighted and presented for the overall sample, as well as separately for individuals who sought informal or formal support. Profiles were also included for individuals experiencing moderate-to-high psychological distress. Help-seeking estimates were age-weighted using the 2000 U.S. standard population with four age groups: 18–24, 25–44, 45–64, and ≥65 years.

Outcomes modeled were (a) any formal or informal help-seeking (Model 1a), (b) seeking informal support (Model 2a), and (c) seeking formal support (Model 3a). To strengthen our findings, a sensitivity analysis was conducted among adults with 30-day moderate-to-high psychological distress seeking any mental health support (formal or informal) (Model 4a), as this subgroup was more likely to seek mental health support. Log-linear regression models were fitted using PROC LOGLINK in SAS-callable SUDAAN 11.0.1 (Research Triangle Institute, Research Triangle Park, NC), which uses a log link and accounts for the complex survey design including sampling weights and stratification. This approach directly estimates prevalence ratios (PR), which are preferred over odds ratios when outcome prevalence is common (>10%), as odds ratios can overestimate the magnitude of associations [29]. PR and 95% confidence intervals (CI) were reported for both the main effects and conditional effects models.

To assess whether the association between help-seeking and social factors differs by race and ethnicity, conditional effects models (1b-4b) incorporated two-way interaction terms of race and ethnicity with gender, socioeconomic status (education, employment, and household poverty levels), as well as immigration-related characteristics (English proficiency, place of birth, and residence duration in the U.S.). Interaction effects were assessed on a multiplicative scale using PRs. For significant interaction terms identified using Wald tests, predicted probabilities were derived from these models to illustrate the direction and magnitude of these effects across subgroups. Post-estimation t-tests were subsequently used to statistically compare these predicted probabilities.

All main effects and interaction models were controlled for lifetime mental health and behavioral health diagnoses, consistent with prior research [30], as individuals with prior diagnoses are more likely to seek support due to ongoing treatment needs. This adjustment helps isolate the association between sociodemographic and immigration-related characteristics and help-seeking, independent of differences in diagnosed condition prevalence across groups.

Missing data ranged from 0.2% to 4.4% in our cohort; as a result, imputation was not conducted, informed by a prior study on handling missing data [31]. Statistical analyses were performed using SAS Enterprise Guide 8.3 (SAS Institute Inc., Cary, NC).

### Ethical considerations

This study received approval from the Institutional Review Board at the City University of New York. Participants were informed about the consent process, and consent was implied through the return of a completed questionnaire by mail or online submission. No signed consent forms were collected. Data collection was conducted between May and September, 2023.

## Results

### Sample characteristics

Of the 43,606 adults (weighted to the NYC adult population, N = 6,924,841; Table 1), 39.3% were aged 25–44. Over half (53.3%) were cisgender women, and the majority (86.3%) identified as straight or heterosexual. A third (33.9%) were white, followed by Hispanic (27.1%) and Black (21.4%). Approximately 40% of participants completed a bachelor’s degree or higher, two-thirds (61.3%) were employed, one-third (33.3%) lived below 200% FPL, and 95.8% had health insurance. In terms of immigration-related characteristics, 59.7% were U.S.-born, 88.6% had English proficiency at well/very well and 91.5% lived in the U.S. for over 10 years or since birth. Regarding lifetime mental and behavioral health diagnoses, 25.6% had received at least one diagnosis in their lifetime, with rates ranging from 1.1% for schizophrenia to 10.7% for major depression/severe depression and 18.1% for anxiety.

**Table 1:** Demographic characteristics among New York City Adults: 2023.

	Among all adults		Among those who sought *informal* support		Among those who sought *formal* support		Among those with past 30-day moderate-to-high psychological distress*	
	n	%	n	%	n	%	n	%
**Total n (%)**	43,606	**100**	14,895	36.2	10,671	24.4	11,190	28.5
**Age (in years)**								
18–24	2,028	10.9	1,036	15.2	534	10.8	819	15.6
25–44	13,599	39.3	6,669	50.3	4,734	51.0	4,408	45.4
45–64	14,438	30.9	4,406	24.2	3,289	26.1	3,500	26.5
≥65	13,541	18.9	2,784	10.3	2,114	12.1	2,463	12.5
**Gender**								
Cisgender man	16,565	45.1	4,739	39.3	3,297	37.8	4,088	43.8
Cisgender woman	26,452	53.3	9,819	57.9	7,040	58.0	6,788	52.6
Other gender identity	589	1.6	337	2.8	334	4.1	314	3.6
Sexual orientation*								
Straight or heterosexual	36,405	86.3	11,925	80.9	8,073	76.0	8,659	78.7
LGB+	4,043	10.1	2,207	15.8	1,954	20.4	1,670	16.4
Not sure	1,238	3.5	396	3.3	325	3.6	460	4.9
**Race and ethnicity**								
White	16,378	33.9	6,601	40.7	4,844	42.4	3,806	29.9
Black	7,985	21.4	2,032	16.3	1,581	17.8	1,689	18.9
Asian	6,900	15.6	2,232	14.9	1,088	9.6	1,998	17.6
Hispanic	10,579	27.1	3,332	25.6	2,674	27.8	3,166	31.2
Other	1,764	2.0	698	2.4	484	2.5	531	2.4
Place of birth*								
Foreign-born	17,022	40.3	4,448	30.1	2,826	26.0	4,287	37.8
U.S.-born	26,301	59.7	10,378	69.9	7,782	74.0	6,838	62.2
English proficiency*								
Not at all well/Not well	4,694	11.4	1,041	7.0	887	7.9	1,479	12.6
Well/Very well	38,729	88.6	13,801	93.0	9,749	92.1	9,659	87.4
Residence duration in the U.S.*								
<5 years	933	3.0	360	3.5	195	2.4	309	3.6
5–10 years	1,795	5.5	609	5.0	367	4.2	550	6.2
>10 years or since birth	40,447	91.5	13,816	91.5	10,013	93.4	10,217	90.2
Marital status*								
Single, never married	13,976	35.3	5,826	43.5	4,479	44.8	4,542	45.8
Married	16,276	41.0	4,684	33.0	2,926	29.9	3,176	29.9
Living with a partner (but not married)	2,986	8.6	1,463	11.7	1,027	11.6	916	9.8
Widowed	3,668	5.0	870	3.2	665	3.8	846	4.2
Divorced or separated	6,334	10.1	1,968	8.6	1,515	10.0	1,632	10.3
**Education level**								
High school or less	11,798	37.5	2,653	26.5	2,277	28.9	3,347	39.9
Some college	9,327	22.1	2,704	20.6	1,924	19.7	2,429	22.8
Bachelor or higher	22,481	40.4	9,538	53.0	6,470	51.4	5,414	37.3
Employment status*								
Employed	24,412	61.3	9,654	66.7	6,233	61.0	6,080	57.7
Unemployed	3,807	10.5	1,579	12.2	1,258	14.0	1,610	17.3
Not in labor force	14,947	28.2	3,561	21.0	3,079	25.0	3,367	24.9
Health insurance*								
Yes	41,296	95.8	14,195	96.5	10,237	97.3	10,446	94.9
No	1,340	4.2	448	3.5	232	2.7	464	5.1
Household poverty levels*								
<200% FPL	14,226	33.3	4,021	28.2	3,243	29.7	4,456	39.7
200–399% FPL	9,640	23.5	2,937	21.4	1,851	19.5	2,423	23.9
≥400% FPL	19,671	43.2	7,911	50.4	5,556	50.8	4,294	36.4
**Lifetime disorders (any)**	11,374	25.6	6,309	40.9	7,247	68.4	5,620	48.2
Major depression	4,728	10.7	2,714	17.9	3,477	33.0	2,993	25.6
Post-traumatic stress disorder	2,467	5.3	1,424	8.9	1,743	16.0	1,448	11.9
Anxiety or generalized anxiety disorder	8,061	18.1	4,745	30.9	5,459	52.4	4,165	35.6
Schizophrenia, schizoaffective disorder, or psychosis	417	1.1	203	1.5	328	3.6	218	2.2
Bipolar disorder, mania, or manic depression	1,179	2.6	643	4.3	880	8.4	716	6.2
Intellectual or developmental disability (including ADHD, autism)	1,362	3.8	891	7.3	927	10.8	774	8.0
Drug use disorder (including cocaine, heroin, etc.)	605	1.4	364	2.4	352	3.4	328	2.9
Alcohol use disorder	840	1.8	473	3.0	443	4.1	426	3.6
**Moderate-to-high psychological distress**	11,190	28.5	5,534	40.3	4,800	49.4	-	-
**12-month help-seeking behaviors**	19,479	46.6	-	-	-	-	7,548	67.7

Due to rounding, the total may not add up to 100% or might exceed it. *Missing <5%. Frequency was unweighted while percentage was weighted.

Sociodemographic profile of individuals who sought informal or formal support was consistent with the overall adult sample. The majority were aged 25–44, cisgender women, and identified as straight or heterosexual. Most participants were white, had a bachelor’s degree or higher, and were employed. Additionally, most were U.S.-born, with high levels of English proficiency and residence in the U.S. for over 10 years.

### Mental health help-seeking and sources of support

**Fig 1:**
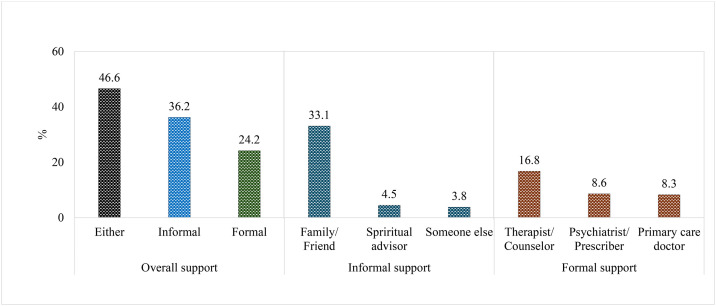
Source of mental health support among New York City adults: 2023.

Overall, 46.6% of adults sought any type of support for issues related to emotions, nerves, or mental health, with 36.2% seeking informal support and 24.2% seeking formal support. Among informal sources, family or friends were most utilized, with 33.1% of the full sample reporting use. Among formal sources, 16.8% of the full sample saw therapists or counselors, with psychiatrists or other prescribers (8.6%) and primary care doctors (8.3%) being less commonly used (Fig 1). Among adults with 30-day moderate-to-high psychological distress, 67.7% sought mental health support (Table 1).

### Main effects models for factors associated with mental health help-seeking

The main effects models included sociodemographic variables, immigration-related characteristics, and lifetime mental and behavioral health diagnoses (Table 2).

**Table 2:** Adjusted prevalence ratios (aPR) and their 95% confidence intervals (CI) for factors associated with mental health help-seeking among New York City adults: 2023.

	Model 1a. Among all adults (n = 43,606)		Model 2a. Among adults who sought *informal* support (n = 14,895)		Model 3a. Among adults who sought *formal* support (n = 10,671)		Model 4a. Among adults with past 30-day moderate-to-high psychological distress (n = 11,190)	
	aPR	95%CI	aPR	95%CI	aPR	95%CI	aPR	95%CI
**Age groups**								
18–24	1.00		1.00		1.00		1.00	
25–44	0.93**	**0.89, 0.98**	0.86***	**0.81, 0.91**	1.23***	**1.13, 1.34**	0.99	0.93, 1.05
45–64	0.78***	**0.74, 0.83**	0.66***	**0.62, 0.71**	1.06	0.96, 1.16	0.93	0.87, 1.00
≥65	0.60***	**0.57, 0.65**	0.49***	**0.45, 0.53**	0.85**	**0.76, 0.94**	0.84***	**0.77, 0.92**
**Gender**								
Cisgender woman	1.00		1.00		1.00		1.00	
Cisgender man	0.84***	**0.81, 0.86**	0.78***	**0.75, 0.81**	0.87***	**0.83, 0.91**	0.91***	**0.88, 0.95**
Other gender identity	0.92*	**0.86, 0.98**	0.91*	**0.82, 1.00**	0.96	0.87, 1.07	1.01	0.95, 1.08
**Sexual orientation**								
Straight or heterosexual	1.00		1.00		1.00		1.00	
LGB+	1.11***	**1.07, 1.14**	1.12***	**1.07, 1.17**	1.20***	**1.13, 1.26**	1.04*	**1.00, 1.09**
Not sure	0.98	0.90, 1.06	0.92	0.83, 1.02	1.12	0.99, 1.27	0.98	0.89, 1.07
**Race and ethnicity**								
White	1.00		1.00		1.00		1.00	
Black	0.88***	**0.85, 0.92**	0.81***	**0.76, 0.86**	0.92*	**0.87, 0.99**	0.96	0.91, 1.01
Asian	0.96	0.92, 1.01	0.97	0.92, 1.03	0.92*	**0.69, 0.81**	0.95	0.90, 1.01
Hispanic	0.96*	**0.93, 1.00**	0.91***	**0.87, 0.95**	0.93**	**0.88, 0.98**	0.95*	**0.90, 0.99**
Other	1.03	0.97, 1.10	1.02	0.95, 1.11	0.98	0.88, 1.09	1.01	0.94, 1.09
**Marital status**								
Married	1.00		1.00		1.00		1.00	
Single and never married	1.17***	**1.13, 1.21**	1.15***	**1.11, 1.20**	1.19***	**1.13, 1.26**	1.03	0.98, 1.07
Living with a partner (but not married)	1.15***	**1.10, 1.21**	1.21***	**1.14, 1.27**	1.12**	**1.04, 1.20**	1.04	0.98, 1.07
Widowed	1.17***	**1.10, 1.26**	1.17**	**1.07, 1.28**	1.15**	**1.05, 1.27**	1.07	0.98, 1.16
Divorced or separated	1.18***	**1.12, 1.23**	1.16***	**1.09, 1.23**	1.20***	**1.12, 1.29**	1.01	0.95, 1.07
**Education level**								
High school or less	1.00		1.00		1.00		1.00	
Some college	1.09***	**1.05, 1.14**	1.19***	**1.12, 1.26**	1.05	0.98, 1.20	1.07*	**1.02, 1.13**
Bachelor or higher	1.31***	**1.26, 1.37**	1.46***	**1.38, 1.54**	1.32***	**1.24, 1.41**	1.15***	**1.09, 1.22**
**Employment status**								
Employed	1.00		1.00		1.00		1.00	
Unemployed	1.09***	**1.04, 1.14**	1.08*	**1.02, 1.14**	1.16***	**1.08, 1.24**	1.05	0.99, 1.10
Not in labor force	1.05*	**1.00, 1.09**	0.98	0.93, 1.04	1.15***	**1.08, 1.22**	1.10**	**1.04, 1.16**
**Household poverty levels**								
<200% FPL	1.00		1.00		1.00		1.00	
200–399% FPL	0.99	0.95, 1.03	1.01	0.96, 1.07	0.98	0.92, 1.05	1.00	0.95, 1.06
≥400% FPL	1.09***	**1.05, 1.14**	1.07**	**1.02, 1.13**	1.16***	**1.09, 1.23**	1.09***	1.04, 1.14
**Health insurance**								
No	1.00		1.00		1.00		1.00	
Yes	0.91*	**0.84, 0.99**	0.92	0.83, 1.01	0.74***	**0.64, 0.86**	0.95	0.87, 1.05
**Place of birth**								
U.S.-born	1.00		1.00		1.00		1.00	
Foreign-born	0.82***	**0.79, 0.85**	0.81***	**0.77, 0.85**	0.82***	**0.77, 0.87**	0.89***	**0.85, 0.94**
**English proficiency**								
Not at all well/not well	1.00		1.00		1.00		1.00	
Well/very well	0.94	0.88, 1.00	1.01	0.92, 1.10	0.83***	**0.75, 0.92**	0.99	0.91, 1.07
**Residence duration in the U.S.**								
>10 years or since birth	1.00		1.00		1.00		1.00	
5–10 years	1.06	0.98, 1.14	1.04	0.95, 1.15	1.07	0.94, 1.22	1.02	0.92, 1.13
<5 years	1.14*	**1.04, 1.24**	1.17**	**1.05, 1.30**	0.97	0.83, 1.12	1.04	0.93, 1.16
**Lifetime disorders**						0.83, 1.12		
Major depression	1.30***	**1.27, 1.34**	1.16***	**1.10, 1.21**	1.63***	**1.55, 1.72**	1.20***	**1.16, 1.24**
Post-traumatic stress disorder	1.10***	**1.06, 1.14**	1.09**	**1.03, 1.15**	1.20***	**1.14, 1.27**	1.05**	**1.01, 1.09**
Anxiety or generalized anxiety disorder	1.60***	**1.56, 1.65**	1.37***	**1.32, 1.43**	1.20***	**2.78, 3.09**	1.31***	**1.26, 1.36**
Schizophrenia, schizoaffective disorder, or psychosis	1.31***	**1.20, 1.43**	1.09	0.94, 1.26	1.61***	**1.39, 1.88**	1.12**	**1.03, 1.21**
Bipolar disorder, mania, or manic depression	1.17***	**1.11, 1.22**	1.11**	**1.03, 1.21**	1.20***	**1.10, 1.30**	1.09***	**1.05, 1.14**
Intellectual or developmental disability (including ADHD, autism)	1.04	0.99, 1.08	1.13***	**1.07, 1.20**	1.14***	**1.07, 1.21**	1.02	0.98, 1.07
Drug use disorder (including cocaine, heroin, etc.)	1.06	0.99, 1.14	1.20**	**1.08, 1.35**	0.97	0.86, 1.09	0.99	0.92, 1.06
Alcohol use disorder	1.10**	**1.03, 1.18**	1.22***	**1.11, 1.34**	1.01	0.91, 1.13	1.06	0.99, 1.13

In Model 1a among all adults, older adults (25–44 years: aPR = 0.93, 95% CI: 0.89, 0.98; 45–64 years: aPR = 0.78, 95% CI: 0.74, 0.83; and ≥65 years: aPR = 0.60, 95% CI: 0.57, 0.65 vs. 18–24 years) and individuals who were not cisgender women (cisgender men: aPR = 0.84, 95% CI: 0.81, 0.86; and other gender identity: aPR = 0.92, 95%CI: 0.86, 0.98 vs. cisgender women) were less likely to seek mental health support. Similarly, adults from minoritized racial and ethnic groups (Black: aPR = 0.88, 95% CI: 0.85, 0.92; and Hispanic: aPR = 0.96, 95% CI: 0.93, 1.00) had lower probabilities of seeking mental health support compared to white adults. LGB+ individuals (aPR = 1.11, 95% CI: 1.07, 1.14) had 1.11 times higher likelihood of help-seeking compared to their straight/heterosexual counterparts. Compared to married individuals, those who were single and never married (aPR = 1.17, 95% CI: 1.13, 1.21), cohabitating without marriage (aPR = 1.15, 95% CI: 1.10, 1.21), widowed (aPR = 1.17, 95% CI: 1.10, 1.26), and divorced or separated (aPR = 1.18, 95% CI: 1.12, 1.23) were more likely to seek support.

Regarding socioeconomic characteristics, higher educational attainment (some college: aPR = 1.09, 95% CI: 1.05, 1.14; bachelor or higher: aPR = 1.31, 95% CI: 1.26, 1.37 vs. high school or less), employment status (unemployed: aPR = 1.09, 95% CI: 1.04, 1.14; not in labor force: aPR = 1.05, 95% CI: 1.00, 1.09 vs. employed) and higher household income levels (≥400% FPL: aPR = 1.09, 95% CI: 1.05, 1.14 vs. < 200% FPL) showed higher probabilities of seeking help. Insured adults were less likely to seek mental health support than uninsured individuals (aPR = 0.91, 95% CI: 0.84, 0.99).

In terms of immigration-related characteristics, place of birth (foreign-born: aPR = 0.82, 95% CI: 0.79, 0.85 vs. U.S.-born) was associated with lower likelihood of help-seeking, while those with shorter residence duration (<5 years: aPR = 1.14, 95% CI: 1.04, 1.24 vs. > 10 years or since birth) had higher probability of seeking support. No significant associations were observed between English proficiency and mental health help-seeking.

Adults with a lifetime mental or behavioral health diagnosis had a significantly higher likelihood of help-seeking in the past 12 months, ranging from 10% higher prevalence for post-traumatic stress disorder (aPR = 1.10, 95% CI: 1.06, 1.14) or alcohol use disorder (aPR = 1.10, 95% CI: 1.03, 1.18) to 60% higher prevalence for anxiety disorders (aPR = 1.60, 95% CI: 1.56, 1.65).

Similar patterns were observed in Model 2a (individuals with informal support) and Model 3a (individuals with formal support). However, health insurance was no longer associated with mental health help-seeking among those who relied on informal support, and residence duration was not associated with help-seeking among those who utilized formal support, but English proficiency became significant. Among those with 30-day moderate-to-high psychological distress (Model 4a), the findings mirrored those of the overall sample (Model 1a), except that marital status, health insurance, and residence duration were not significantly associated with help-seeking (Table 2).

### Conditional effects models for factors associated with mental health help-seeking

The conditional effects models included all covariates from the main effects models, along with two-way interaction terms between race and ethnicity with gender, socioeconomic status (education, employment, and household poverty levels), and immigration-related characteristics (place of birth, English proficiency, and length of residence) (Table 3).

**Table 3:** Two-way interactions of race and ethnicity with gender, socioeconomic status, and immigration-related characteristics on mental health help-seeking among New York City adults: 2023.

	Model 1b. Among all adults(n=43,606)		Model 2b. Among adults who sought *informal* support (n=14,895)		Model 3b. Among adults who sought *formal* support (n=10,671)		Model 4b. Among adults with past 30-day moderate-to-high psychological distress (n=11,190)	
	aPR	95%CI	aPR	95%CI	aPR	95%CI	aPR	95%CI
**Age groups**								
18–24	1.00		1.00		1.00		1.00	
25–44	0.93**	**0.89, 0.98**	0.86***	**0.81, 0.91**	1.23***	**1.13, 1.34**	0.99	0.93, 1.05
45–64	0.78***	**0.74, 0.83**	0.67***	**0.62, 0.71**	1.06	0.96, 1.16	0.94	0.87, 1.00
≥65	0.61***	**0.58, 0.66**	0.50***	**0.46, 0.55**	0.87*	**0.78, 0.97**	0.85***	**0.78, 0.93**
**Gender**								
Cisgender woman	1.00		1.00		1.00		1.00	
Cisgender man	0.84***	**0.81, 0.88**	0.78***	**0.75, 0.81**	0.83***	**0.78, 0.88**	0.91***	**0.88, 0.95**
Other gender identity	0.88**	**0.81, 0.96**	0.91*	**0.83, 1.00**	0.91	0.78, 1.06	1.01	0.95, 1.08
**Sexual orientation**								
Straight or heterosexual	1.00		1.00		1.00		1.00	
LGB+	1.11***	**1.07, 1.15**	1.12***	**1.07, 1.17**	1.20***	**1.14, 1.27**	1.05*	**1.00, 1.09**
Not sure	0.98	0.90, 1.06	0.92	0.83, 1.02	1.12	0.98, 1.27	0.98	0.89, 1.08
**Race and ethnicity**								
White	1.00		1.00		1.00		1.00	
Black	0.73	0.47, 1.14	0.70	0.39, 1.27	0.91	0.82, 1.00	0.73	0.41, 1.29
Asian	0.80*	**0.66, 0.97**	0.73*	**0.58, 0.94**	0.72***	**0.64, 0.82**	0.86	0.68, 1.08
Hispanic	0.63***	**0.54, 0.75**	0.54***	**0.43, 0.68**	0.85***	**0.79, 0.92**	0.71**	0.68, 1.08
Other	0.63	0.27, 1.15	0.63	0.28, 1.39	0.85	0.72, 1.00	1.24	0.90, 1.70
**Marital status**								
Married	1.00		1.00		1.00		1.00	
Single and never married	1.16***	**1.12, 1.20**	1.15***	**1.10, 1.20**	1.18***	**1.12, 1.24**	1.03	0.98, 1.08
Living with a partner (but not married)	1.16***	**1.10, 1.21**	1.21***	**1.14, 1.28**	1.11**	**1.12, 1.24**	1.04	0.98, 1.10
Widowed	1.16***	**1.09, 1.24**	1.17**	**1.06, 1.28**	1.15**	**1.04, 1.27**	1.06	0.98, 1.15
Divorced or separated	1.18***	**1.12, 1.23**	1.16***	**1.10, 1.24**	1.19***	**1.11, 1.28**	1.01	0.95, 1.08
**Education level**								
High school or less	1.00		1.00		1.00		1.00	
Some college	1.10***	**1.05, 1.15**	1.19***	**1.12, 1.27**	1.06	0.99, 1.13	1.08**	**1.02, 1.14**
Bachelor or higher	1.32***	**1.26, 1.37**	1.46***	**1.38, 1.55**	1.33***	**1.25, 1.42**	1.16***	**1.10, 1.22**
**Employment status**								
Employed	1.00		1.00		1.00		1.00	
Unemployed	1.04	0.98, 1.11	1.02	0.94, 1.10	1.08	0.98, 1.19	1.01	0.93, 1.08
Not in labor force	0.93*	**0.88, 0.99**	0.86***	**0.80, 0.93**	1.00	0.92, 1.08	0.97	0.90, 1.05
**Household poverty levels**						0.92, 1.08		
<200% FPL	1.00		1.00		1.00		1.00	
200–399% FPL	1.00	0.96, 1.04	1.02	0.97, 1.08	0.99	0.92, 1.05	1.01	0.96, 1.06
≥400% FPL	1.10***	**1.06, 1.14**	1.08**	**1.03, 1.14**	1.16***	**1.10, 1.23**	1.09***	**1.04, 1.15**
**Health insurance**								
No	1.00		1.00		1.00		1.00	
Yes	0.91*	**0.84, 0.99**	0.92	0.84, 1.02	0.74***	**0.64, 0.86**	0.96	0.87, 1.05
**Place of birth**								
U.S.-born	1.00		1.00		1.00		1.00	
Foreign-born	0.90***	**0.86, 0.96**	0.87***	**0.81, 0.93**	0.91*	**0.83, 0.99**	0.94	0.87, 1.02
**English proficiency**								
Not at all well/not well	1.00		1.00		1.00		1.00	
Well/very well	0.73***	**0.64, 0.83**	0.76**	**0.64, 0.90**	0.85**	**0.77, 0.94**	0.88	0.76, 1.02
**Length of stay in the USA**								
>10 years or since birth	1.00		1.00		1.00		1.00	
5–10 years	1.06	0.98, 1.14	1.05	0.95, 1.15	1.07	0.94, 1.22	1.03	0.92, 1.14
<5 years	1.12*	**1.03, 1.22**	1.15*	**1.03, 1.28**	0.95	0.82, 1.10	1.03	0.92, 1.15
**Lifetime disorders**								
Major depression	1.30***	**1.26, 1.34**	1.15***	**1.10, 1.21**	1.63***	**1.55, 1.71**	1.20***	**1.16, 1.24**
Post-traumatic stress disorder	1.10***	**1.07, 1.14**	1.09**	**1.03, 1.15**	1.21***	**1.14, 1.27**	1.06**	**1.02, 1.10**
Anxiety or generalized anxiety disorder	1.60***	**1.56, 1.64**	1.37***	**1.32, 1.42**	2.92***	**2.77, 3.08**	1.31***	**1.26, 1.35**
Schizophrenia, schizoaffective disorder, or psychosis	1.31***	**1.20, 1.42**	1.08	**1.32, 1.42**	1.61***	**1.39, 1.87**	1.11**	**1.03, 1.21**
Bipolar disorder, mania, or manic depression	1.16***	**1.10, 1.21**	1.10*	**1.02, 1.19**	1.19***	**1.39, 1.87**	1.08***	**1.04, 1.13**
Intellectual or developmental disability (including ADHD, autism)	1.04	1.00, 1.09	1.14***	**1.07, 1.21**	1.14***	**1.07, 1.21**	1.03	0.98, 1.07
Drug use disorder (including cocaine, heroin, etc.)	1.06	0.99, 1.14	1.20**	**1.08, 1.34**	0.96	0.86, 1.08	0.99	0.92, 1.06
Alcohol use disorder	1.11***	**1.04, 1.19**	1.23***	**1.12, 1.35**	1.02	0.92, 1.14	1.07	0.99, 1.14
**Overall p-value for all interaction terms**		**<0.001**		**<0.001**		**<0.001**		**<0.001**
**Race and ethnicity with gender**		**0.003**				**0.001**		
White, cisgender woman	1.00				1.00			
Black, cisgender man	0.97	0.88, 1.06			1.02	0.89, 1.18		
Black, other identity	1.17	0.94, 1.46			1.02	0.72, 1.45		
Asian, cisgender man	0.87**	0.94, 1.46			1.01	0.87, 1.17		
Asian, other identity	1.13	0.93, 1.38			1.72***	**1.29, 2.28**		
Hispanic, cisgender man	1.05	0.98, 1.12			1.13*	**1.02, 1.26**		
Hispanic, other identity	1.04	0.91, 1.19			1.06	0.85, 1.33		
Other, cisgender man	1.14	1.00, 1.30			1.23	0.98, 1.55		
Other, other identity	1.12	0.86, 1.46			0.94	0.70, 1.26		
**Race and ethnicity with employment**		**<0.001**		**<0.001**		**0.005**		**0.001**
White, employed	1.00		1.00		1.00		1.00	
Black, unemployed	1.12*	1.00, 1.26	1.16	0.99, 1.35	1.20*	**1.01, 1.43**	1.05	0.92, 1.20
Black, not in labor force	1.14**	**1.03, 1.25**	1.09	0.96, 1.25	1.21**	**1.05, 1.39**	1.08	0.95, 1.22
Asian, unemployed	1.08	0.94, 1.23	1.13	0.96, 1.33	1.15	0.92, 1.43	1.11	0.95, 1.30
Asian, not in labor force	1.14*	**1.02, 1.28**	1.26**	**1.10, 1.44**	1.29**	**1.08, 1.53**	1.20*	**1.05, 1.38**
Hispanic, unemployed	1.03	0.93, 1.14	1.03	0.90, 1.18	1.08	0.93, 1.26	1.05	0.94, 1.18
Hispanic, not in labor force	1.25***	**1.15, 1.35**	1.31***	**1.17, 1.46**	1.26***	**1.12, 1.41**	1.25***	**1.12, 1.38**
Other, unemployed	1.01	0.83, 1.21	1.07	0.83, 1.38	1.26***	0.79, 1.43	0.95	0.77, 1.19
Other, not in labor force	1.12	0.94, 1.31	1.21	0.98, 1.50	1.21	0.93, 1.57	0.97	0.83, 1.12
**Race and ethnicity with place of birth**		**<0.001**		**<0.001**		**0.002**		**0.018**
White, U.S.-born	1.00		1.00		1.00		1.00	
Black, foreign-born	0.70***	**0.63, 0.79**	0.70***	**0.61, 0.81**	0.73***	**0.62, 0.87**	0.77**	**0.66, 0.91**
Asian, foreign-born	0.94	0.85, 1.03	0.97	0.87, 1.09	0.85*	**0.73, 0.99**	0.95	0.84, 1.08
Hispanic, foreign-born	0.95	0.87, 1.03	0.98	0.88, 1.09	0.91	0.81, 1.03	0.99	0.88, 1.11
Other, foreign-born	0.98	0.84, 1.14	1.07	0.88, 1.30	0.91	0.85, 1.41	1.08	0.91, 1.30
**Race and ethnicity with English proficiency**		**<0.001**		**<0.001**				**0.008**
White, not at all well/not well	1.00		1.00				1.00	
Black, well/very well	1.27	0.82, 1.97	1.21	0.68, 2.18			1.37	0.78, 2.43
Asian, well/very well	1.23*	**1.03, 1.47**	1.23	0.68, 2.18			1.05	0.85, 1.29
Hispanic, well/very well	1.47***	**1.25, 1.72**	1.64***	**1.32, 2.03**			1.29**	**1.06, 1.55**
Other, well/very well	1.73	0.85, 3.51	1.56	0.71, 3.42			0.82	0.60, 1.10

Models were adjusted for age, gender, race and ethnicity, sexual orientation, marital status, education, employment, household poverty, insurance, place of birth, English proficiency, and residence duration.

For Model 1b seeking formal or informal support, significant interaction terms were found between race and ethnicity with gender (p = 0.003), employment status (p < 0.001), place of birth (p < 0.001), and English proficiency (p < 0.001; Table 3). Men from all racial and ethnic groups, except the ‘other’ group—white (45.4% vs. 53.9%, p < 0.001), Black (37.9% vs. 46.5%, p < 0.001), Asian (39.3% vs. 53.3%, p < 0.001), and Hispanic (44.6% vs. 50.6%, p < 0.001)—were less likely to seek mental health support than women. Compared to employed Black adults (41.4%), unemployed Black individuals (48.3%, p = 0.003) were more likely to seek mental health support. This was not the case for other races or ethnicities. U.S.-born adults from all racial and ethnic groups, except other—white (51.7% vs. 46.7%, p < 0.001), Black (48.8% vs. 31.1% p < 0.001), Asian (49.7% vs. 42.0%, p < 0.001), and Hispanic (50.2% vs. 43.0%, p < 0.001)—were more likely to seek mental health support than their foreign-born counterparts. White adults who spoke English well or very well were less likely to seek help compared to white individuals with limited English proficiency (48.7% vs. 66.5%, p < 0.001) but there was no difference for other racial or ethnic groups (Table 4).

**Table 4:** Predicted probabilities (%) of mental health help-seeking by race and ethnicity with intersecting characteristics among New York City adults: 2023.

Panel A: Race and ethnicity with gender						
**Model**	**Gender**	**White**	**Black**	**Asian**	**Hispanic**	**Other**
Any support	Cisgender man	45.4	37.9	39.3	44.6	49.2
	Cisgender woman	53.9	46.5	53.3	50.6	51.2
	Other gender identity	47.3	48	52.9	46.1	50.3
	p-value (man vs. woman)	**<0.001**	**<0.001**	**<0.001**	**<0.001**	0.541
	p-value (other vs. woman)	**0.001**	0.775	0.938	0.099	0.889
Informal support	—	—	—	—	—	—
Formal support	Cisgender man	23.7	21.8	17.9	23.7	26.8
	Cisgender woman	28.5	25.6	21.4	25.2	26.2
	Other gender identity	26.0	23.8	33.5	24.4	22.5
	p-value (man vs. woman)	**<0.001**	**0.009**	**0.009**	0.173	0.832
	p-value (other vs. woman)	0.211	0.647	**0.004**	0.716	0.226
**Panel B: Race and ethnicity with employment status**						
**Model**	**Employment status**	**White**	**Black**	**Asian**	**Hispanic**	**Other**
Any support	Employed	50.4	41.4	45.8	45.7	49.5
	Unemployed	52.5	48.3	51.4	49.1	51.8
	Not in labor force	47	43.9	48.8	53.2	51.7
	p-value (unemployed vs. employed)	0.227	**0.003**	0.069	0.083	0.621
	p-value (not in labor force vs. employed)	**0.015**	0.183	0.229	**<0.001**	0.578
Informal support	Employed	41.1	31.4	36.4	35.2	39.8
	Unemployed	41.9	37.1	41.8	37.1	43.3
	Not in labor force	35.3	29.5	39.3	39.5	41.4
	p-value (unemployed vs. employed)	0.671	**0.015**	0.073	0.352	0.507
	p-value (not in labor force vs. employed)	**<0.001**	0.316	0.219	**0.017**	0.69
Formal support	Employed	26.1	22.1	18.7	22.8	24.7
	Unemployed	28.1	28.6	23.1	26.5	28.3
	Not in labor force	26.1	26.7	24.1	28.6	29.8
	p-value (unemployed vs. employed)	0.137	**0.002**	0.054	**0.02**	0.361
	p-value (not in labor force vs. employed)	0.994	**0.005**	**0.004**	**<0.001**	0.175
**Panel C: Race and ethnicity with place of birth**						
**Model**	**Place of birth**	**White**	**Black**	**Asian**	**Hispanic**	**Other**
Any support	Foreign-born	46.7	31.1	42.0	43.0	46.4
	U.S.-born	51.7	48.8	49.7	50.2	52.2
	p-value	**<0.001**	**<0.001**	**<0.001**	**<0.001**	0.097
Informal support	Foreign-born	36.5	22.4	33.7	32.6	38.6
	U.S.-born	41.9	36.5	39.7	38.1	41.5
	p-value	**<0.001**	**<0.001**	**0.001**	**<0.001**	0.427
Formal support	Foreign-born	24.6	17.7	17.0	21.5	26.1
	U.S.-born	27.1	26.7	21.9	25.9	26.4
	p-value	**0.02**	**<0.001**	**<0.001**	**<0.001**	0.943
**Panel D: Race and ethnicity with English proficiency**						
**Model**	**English proficiency**	**White**	**Black**	**Asian**	**Hispanic**	**Other**
Any support	Not at all well/Not well	66.5	45.8	51.8	44.7	40.3
	Very well/Well	48.7	42.6	46.8	48.0	51
	p-value	**<0.001**	0.746	0.117	0.135	0.459
Informal support	Not at all well/Not well	51.7	34.3	40.1	29.7	34.7
	Very well/Well	39.2	31.5	37.4	36.8	41
	p-value	**0.005**	0.776	0.384	**0.001**	0.648
Formal support	—	—	—	—	—	—

Note: Predicted probabilities (%) were derived from Models 1b-3b. “—” indicates the interaction was not significant for that model and is therefore not presented. P-values are from post-estimation t-tests. Bold p-values indicate statistical significance at p < 0.05.

For Model 2b (individuals seeking informal support), findings were similar to Model 1b, but no interaction was found between race and ethnicity with gender. U.S.-born from all racial and ethnic groups, except other, were consistently more likely to seek informal help compared to foreign-born individuals (Table 3). While white adults who spoke English well or very well were less likely to seek informal mental health support compared to white individuals with limited English proficiency (39.2% vs. 51.7%, p = 0.005), the opposite was true for Hispanic individuals. Hispanic adults with high English proficiency were more likely to seek informal support (36.8% vs. 29.7%, p = 0.001). Compared to employed white adults (41.1%), those not in the labor force (35.3%, p < 0.001) were less likely to seek informal support. In contrast, unemployed Black adults (37.1% vs. 31.4%, p = 0.015) and Hispanic adults not in the labor force (39.5% vs. 35.2%, p = 0.017) were more likely to seek informal help compared to their employed counterparts, respectively (Table 4).

For Model 3b (individuals seeking formal support), it mirrored the findings for Model 1b, except no significant interaction found between race and ethnicity with English proficiency (Table 3). U.S.-born adults from white (27.1% vs. 24.6%, p = 0.020), Black (26.7% vs. 17.7%, p < 0.001), Asian (21.9% vs. 17.0%, p < 0.001), and Hispanic (25.9% vs. 21.5%, p < 0.001) groups, as well as women from white (28.5% vs. 23.7%, p < 0.001), Black (25.6% vs. 21.8%, p = 0.009), and Asian (21.4% vs. 17.9%, p = 0.009) groups, sought more formal help compared to their foreign-born counterparts and men, respectively. Similarly, unemployed Black (28.6% vs. 22.1%, p = 0.002) and Hispanic (26.5% vs. 22.8%, p = 0.02) adults were more likely to seek formal support compared to their employed counterparts (Table 4).

For Model 4b (individuals with past 30-day moderate-to-high psychological distress seeking either formal or informal support), interactions were observed between race and ethnicity with employment status, English proficiency, and place of birth, but not gender (Table 3). Particularly, Asian (73.4% vs. 62.7%, p = 0.017) and Hispanic (76.7% vs. 63.2%, p < 0.001) adults who were not in labor force were more likely to seek mental health support compared to their employed counterparts. U.S.-born Black (71.7% vs. 52.3%, p < 0.001) and Asian (68.8% vs. 61.7%, p = 0.046) adults sought support more than their foreign-born peers. Among Hispanic adults, those with very good/good English proficiency were more likely to seek mental health support than those with limited English proficiency (67.5% vs. 59.7%, p = 0.045; Table 5).

**Table 5:** Predicted probabilities (%) of any mental health help-seeking by race and ethnicity with intersecting characteristics among adults with past 30-day moderate-to-high psychological distress in New York City: 2023.

Panel A: Race and ethnicity with employment status					
**Employment status**	**White**	**Black**	**Asian**	**Hispanic**	**Other**
Employed	70.8	64.1	62.7	63.2	75.3
Unemployed	71.2	67.6	70.2	66.7	72.2
Not in labor force	68.9	67.4	73.4	76.7	70.7
p-value (unemployed vs. employed)	0.882	0.376	0.118	0.263	0.694
p-value (not in labor force vs. employed)	0.503	0.371	**0.017**	**<0.001**	0.364
**Panel B: Race and ethnicity with place of birth**					
**Place of birth**	**White**	**Black**	**Asian**	**Hispanic**	**Other**
Foreign-born	67.7	52.3	61.7	63.6	74.8
U.S.-born	71.8	71.7	68.8	68.3	73.2
p-value	0.133	**<0.001**	**0.046**	0.114	0.797
**Panel C: Race and ethnicity with English proficiency**					
**English proficiency**	**White**	**Black**	**Asian**	**Hispanic**	**Other**
Not at all well/Not well	79.2	54.9	71.4	59.7	99.1
Very well/Well	69.6	66.3	65.9	67.5	71.1
p-value	0.098	0.461	0.306	**0.045**	**0.033**

Note: Predicted probabilities (%) were derived from Model 4b. P-values are from post-estimation t-tests. Bold p-values indicate statistical significance at p < 0.05.

## Discussion

Our study examined mental health help-seeking, including both formal and informal support within a diverse urban city. We found that a sizable proportion of NYC adults sought mental health support, with informal sources, particularly family or friend, being the most prevalent. Several factors were associated with a higher likelihood of seeking support among all adults, including demographic characteristics (younger age, being a woman, identifying as LGB +, being white, and being unmarried), socioeconomic status (higher educational attainment, unemployment, lack of insurance, and higher income), immigration-related factors (being U.S.-born and having a shorter residence duration in the U.S.), and having a lifetime diagnosis of a mental or behavioral health condition. Additionally, the relationship between any mental health help-seeking and factors such as gender, employment status, place of birth, and English proficiency differed across racial and ethnic groups. These effects were also seen for both formal and informal support with two exceptions: gender differences did not vary by race or ethnicity for informal support, and English proficiency did not show differences across racial and ethnic groups for formal support. The findings among individuals with past 30-day moderate-to-high psychological distress mirrored those of the full sample, supporting that the overall patterns were consistent and reliable.

Overall, 46.6% of all adults and 67.7% of adults with past 30-day moderate-to-high psychological distress in our cohort sought mental health support. While U.S. comparisons were not available, these rates appear higher than those reported in Switzerland (35.2%) [5] and Ethiopia (42.2%) [32], likely due to differences in study population and methods. Over one-third of NYC adults turned to informal sources of help, such as family or friends, while just under one-quarter used formal support from mental health professionals. These patterns suggest that informal networks continue to play a critical role in how individuals cope with mental health challenges, potentially due to accessibility, affordability, or cultural acceptability [33]. While not all individuals may require professional care, the greater reliance on informal support may suggest ongoing barriers to professional mental health care for some individuals, including cost, stigma, and lack of culturally appropriate services [34]. This concern is particularly relevant in NYC, where 60% of NYC residents are enrolled in Medicaid or Essential Plan, and many now risk losing coverage due to newly-implemented eligibility and verification requirements [35].

In this study, the relationship between mental health help-seeking and gender, employment status, place of birth, and English proficiency varied across racial and ethnic groups. In contrast, no significant interactions were observed between race and ethnicity and age, sexual orientation, marital status, educational attainment, household poverty levels, insurance status, or residence duration in the U.S. These results show how race and ethnicity can interact with other social characteristics to influence mental health help-seeking, meaning that multiple identities need to be considered in both research and the design of mental health interventions. Particularly, cisgender men were less likely than cisgender women to seek mental health support across most racial and ethnic groups, except those identifying as other race, reaffirming long-standing gender disparities in help-seeking. This pattern held true for both any and formal support-seeking, likely reflecting the influence of social expectations that discourage men from expressing emotional distress or seeking professional help [36]. However, gender differences were less pronounced with informal help-seeking in this cohort. These findings align with prior investigation suggesting that men are similarly likely as women to seek informal help, but less likely to seek professional care, especially for conditions like depression [37]. Our results underscore the need to raise awareness about the barriers men face in seeking formal mental health support and the importance of challenging societal norms that perpetuate these disparities. Mental health outreach should be tailored by both gender and race/ethnicity. Particularly, health promotion programs for men may be more effective when embedded within male-relevant settings, such as sport clubs and workplaces that ease participation and reduce stigma [38]. For example, barbershop-based health promotion interventions have shown promise as culturally aligned platforms for mental health outreach among Black men [39].

Employment status interacted with race and ethnicity to influence mental health help-seeking, most notably among Black adults. This may be due to the disproportionate economic impact of unemployment in Black communities [40], where structural inequities such as racialized wealth gaps and job discrimination can worsen the psychological toll of job loss. In our study, unemployed Black adults were more likely to seek mental health support than their employed counterparts, suggesting that job loss or financial instability may increase distress levels, leading to motivation to seek help [41]. This pattern was seen for both informal and formal mental health help-seeking among Black adults, signaling the complex ways employment and cultural factors shape mental health support-seeking across racial and ethnic groups. These findings suggest that embedding mental health resources within employment and workforce development services [42], particularly in communities with large Black populations, could capitalize on the increased willingness to seek help during periods of job loss.

Across all racial and ethnic groups, foreign-born adults were consistently less likely than U.S.-born adults to seek mental health support, both formal and informal, except for those categorized as other race. Our findings are congruent with existing literature on immigrant health disparities and underscores the ongoing impact of structural and cultural barriers. These may include language challenges, unfamiliarity with the healthcare system, stigma within immigrant communities, and fear of jeopardizing immigration status [20,43]. The consistency of this finding across diverse groups emphasizes the urgent need for culturally tailored outreach and immigrant-inclusive mental health services, especially in NYC, which is often described as the quintessential city of immigrants. Promising strategies include deploying community health workers, who have been shown to improve mental health outcomes among immigrant populations [44], and partnering with trusted faith-based organizations and community centers as entry points for mental health support [45].

The relationship between English proficiency and mental health help-seeking varied across racial and ethnic groups. Among white adults, those with high English proficiency were less likely to seek any support and through informal sources, though this pattern did not apply to formal support. One possible explanation is that higher English proficiency may be linked to greater confidence in managing daily life, potentially reducing reliance on informal networks [20]; however, cultural values may also play a role. In some white cultural contexts, there may be a stronger emphasis on independence [46], which can discourage sharing mental health struggles with family and friends. In contrast, Hispanic adults with higher English proficiency were more likely to seek informal support than those with limited proficiency. This may reflect cultural norms like familismo, which values strong family ties and interdependence [47]. Better English fluency may improve access to support networks, increase mental health literacy, and reduce stigma in help-seeking [48], enabling Hispanic adults to better engage their families and communities when needed. Accordingly, language access initiatives alone are insufficient; they must be paired with culturally specific engagement strategies, such as peer-delivered and family-centered interventions delivered in individuals’ preferred language [49,50].

In this study, higher education, shorter residence duration, and higher income were associated with a greater likelihood of seeking any mental health support, which are supported by previous investigations [19,51]. However, these factors did not differ by race and ethnicity, suggesting that their influence on help-seeking behaviors may be relatively consistent across racial and ethnic groups. This finding shows that while these social determinants play an important role in shaping access to and use of mental health support, their effects may not be strongly moderated by racial or ethnic identity. It also indicates the value of examining both independent and intersecting effects of social factors, as not all variables interact to produce differential outcomes across groups.

Our study has several notable strengths. First, the use of address-based sampling enabled a more representative sample of the adult population, including individuals who are often hard to reach through conventional survey methods. Second, the large sample size allowed for robust analyses, including the exploration of interactions across multiple sociodemographic and immigration-related characteristics with race and ethnicity. Third, by capturing both informal and formal help-seeking, the study offers a more comprehensive understanding of how individuals engage with different sources of mental health support. However, several limitations should be considered. A key limitation of our study is that, due to its cross-sectional design, we cannot infer causality from the observed associations. Additionally, the study relies on self-reported data regarding mental health help-seeking in the past 12 months, which may be subject to recall bias. Residual confounding from unmeasured variables such as perceived stigma cannot be ruled out. Despite address-based sampling, certain populations such as individuals experiencing homelessness or undocumented immigrants may be underrepresented, as individuals with immigration concerns may have been reluctant to participate. Given NYC’s exceptional demographic diversity and urban infrastructure, findings may not generalize to less diverse or rural settings. Finally, while we examined interactions between race and ethnicity and other social identities, we did not explore interactions between gender and other identities or three-way interactions (e.g., race and ethnicity, gender, and employment). Future research should address these aspects to better understand the factors influencing mental health help-seeking behavior.

## Conclusion

This study provides valuable insights into the patterns and factors associated with mental health help-seeking in NYC, offering a comprehensive examination of both informal and formal support sources. Nearly half of adults sought any support, with family or friends being the most common informal sources, highlighting the importance of recognizing and strengthening these trusted networks alongside formal mental health services. Mental health help-seeking varied significantly by sociodemographic and immigration-related factors. Several characteristics were associated with a higher likelihood of seeking any help, including younger age, being female, white, LGB +, unmarried, more educated, higher-income, U.S.-born, or having a lifetime mental and behavioral health diagnosis. Notably, associations between any mental health help-seeking and key factors, such as gender, employment status, place of birth, and English proficiency, differed across racial and ethnic groups. Specifically, white adults with high English proficiency and employed Black adults were less likely to seek help compared to white adults with limited English proficiency and unemployed Black adults. These findings demonstrate that mental health help-seeking does not follow a single pattern. Interventions should therefore be flexible and culturally responsive, addressing specific factors associated with lower mental health help-seeking. Future research should explore the three-way interactions between these factors to further clarify how overlapping identities shape mental health help-seeking.
